# Impact of pre-treatment in GnRH-antagonist cycles triggered with GnRH agonist on reproductive outcomes

**DOI:** 10.5935/1518-0557.20230022

**Published:** 2024

**Authors:** Einat Zivi, Talia Eldar-Geva, Esther Rubinstein, Nava Dekel, Oshrat Schonberger, Ido Ben-Ami

**Affiliations:** 1 IVF and infertility Unit, Department of Obstetrics and Gynecology, Shaare Zedek Medical Center (affiliated with the Hebrew University School of Medicine) Jerusalem, Israel

**Keywords:** pre-treatment, GnRH-agonist, hormonal contraception, maturation response, pituitary suppression

## Abstract

**Objective:**

Pre-treatment (PT) therapies in IVF are known to be used as pre-stimulation modality to improve cycle outcomes. This study aims to assess whether PT in GnRH antagonist cycles triggered with GnRH-agonist impact oocyte maturation response.

**Methods:**

Data were retrospectively collected for patients who underwent GnRH antagonist cycle with agonist triggering with and without PT. The patients were allocated to groups according to their PT status. The primary outcome evaluated was suboptimal maturation response. Suboptimal maturation to trigger was defined as no oocyte upon retrieval when adequate response was expected.

**Results:**

The study population included 196 patients who underwent GnRH antagonist cycle with agonist triggering. The study group included 69 patients who received PT. The control group included 127 patients with no PT. In univariate analysis, the PT group significantly displayed suboptimal response compared to the controls (*p* = 0.008). All the patients in the study group with suboptimal response (with or without hCG re-triggering) were treated with GnRH-agonist as PT. Basal and pre-trigger LH values were significantly lower in the study group compared to controls (*p* < 0.001). Multivariate regression analysis revealed that PT with GnRH agonist was a significant predictor for suboptimal response.

**Conclusions:**

Pre-treatment, and particularly the use of GnRH-agonist as PT in antagonist cycles triggered with agonist, increases the risk of suboptimal response to GnRH-agonist trigger. This might be explained by prolonged pituitary suppression, which lasts beyond the PT cessation.

## INTRODUCTION

Pre-treatment therapies in IVF protocols are defined as hormonal interventions before the beginning of gonadotropin stimulation. They are mostly used in the context of GnRH-antagonist protocol cycles. These therapies aim to induce synchronization of follicular development and enable IVF cycle scheduling by reducing LH and/or FSH secretion prior to stimulation. There are several types of PT: estrogen or progesterone supplementation, combined hormonal contraceptive (CHC) methods (oral, transdermal or vaginal), GnRH antagonist administration with or without estrogen priming, and GnRH agonists.

The literature has been conflicting in their overall efficacy regarding live births, pregnancy rates and number of mature oocytes retrieved. Furthermore, the safety of pre-treatment modalities was recently issued in the European Society of Human Reproduction and Embryology (ESHRE) guidelines on ovarian stimulation for IVF/ICSI (The ESHRE Guideline Group on Ovarian Stimulation, 2020). According to a meta-analysis (Farquhar *et al*., 2017) cited in the guideline, in cases of the GnRH antagonist protocol, in which oral contraceptive pills (OCPs) are used as PT, the rates of live births and ongoing pregnancies were lower than in cases of no PT. Hence, the ESHRE guideline recommended avoiding OCP as PT. More recent studies published on this matter have shown different results (Montoya-Botero *et al*., 2020; Lu *et al*., 2020).

In addition to the aforementioned reproductive outcomes, PT with OCP has also been reported to result in suboptimal oocyte yield in agonist-triggered cycles (Popovic-Todorovic *et al*., 2019; Meyer *et al*., 2015). This effect has been attributed to prolonged pituitary desensitization caused by long-term OCP administration. Notably, regardless of OCP administration, pituitary suppression by itself is considered a risk factor for suboptimal response (Popovic-Todorovic *et al*., 2019; Meyer *et al*., 2015; Chang *et al*., 2016; Lu *et al*., 2016).

An additional PT therapy used along with the GnRH antagonist protocol is the GnRH agonist. The combined protocol is known as the “agonist-antagonist protocol”. In the agonist-antagonist protocol, mid-luteal administration of GnRH-agonist achieves pituitary desensitization prior to stimulation. The agonist is stopped in the early follicular phase and replaced by gonadotropin stimulation. GnRH antagonist is added during stimulation to prevent a premature LH surge. To our knowledge, no publications have yet reported on the reproductive outcomes of agonist pre-stimulation in GnRH antagonist cycles triggered with agonists.

We conducted an observational, retrospective cohort study in which we assessed the impact of PT on IVF stimulation outcomes. Our study population included patients undergoing IVF cycles for various clinical indications. The patients were under a standard antagonist protocol, with or without PT, triggered by an agonist for final oocyte maturation.

The primary outcome investigated in this study was the presence of suboptimal maturation response. Unsatisfying response to triggering has many possible definitions in the literature. Most studies define it according to retrieval outcomes such as less than the 2.5^th^ to the 10^th^ percentile of oocyte yield (Popovic-Todorovic *et al*., 2019; Shapiro *et al.,* 2011), a cycle requiring re-triggering with hCG (Chang *et al*., 2016), the failure to recover oocytes from the first aspirated follicles (Meyer *et al*., 2015; Chang *et al*., 2016) and the extreme scenario of empty follicle syndrome (EFS), in which there is failure to retrieve any oocytes (Castillo *et al*., 2012). Some studies have considered post-trigger but pre-retrieval parameters, such as LH and progesterone levels, in defining suboptimal response (Popovic-Todorovic *et al*., 2019; Meyer *et al*., 2015; Chang *et al*., 2016).

In accordance with the studies mentioned above, we defined a suboptimal maturation response as no oocyte at retrieval when adequate response was expected according to follicular development and estradiol levels during stimulation. This definition also included cases in which hCG re-triggering was indicated.

Since prolonged pituitary suppression might impair matured oocyte yield in GnRH agonist-triggered cycles, we aimed to assess whether PT, and especially PT using GnRH agonist, had the same impact.

## MATERIALS AND METHODS

### Patients and study design

The retrospective dataset included a total of 196 patients undergoing GnRH antagonist cycles triggered solely with GnRH agonists, performed between 2019 and 2021. The study protocol was approved by the local ethics committee (0388-20-SZMC). The requirement for informed consent was waived due to the observational nature of the study.

The study group included patients who were treated prior to the beginning of stimulation with either CHC, midluteal estrogen or progesterone supplementation, GnRH antagonist with estrogen priming or mid-luteal GnRH agonist administration (Decapeptyl 0.1-mg per day). The control group included patients who did not receive PT.

We analyzed the clinical and laboratory data of patients. Details about patient age, BMI, ovarian reserve parameters and cause of referral to the IVF clinic were collected from the patients’ medical histories, as recorded in their personal files. Information on the use of pre-treatment, stimulation cycle parameters (the number and size of follicles assessed by transvaginal ultrasound and serum estradiol, progesterone and LH during ovarian stimulation), the number of oocytes retrieved and the oocyte maturation rate were all gathered from the hospital electronic medical records and the patient’s personal file.

The primary outcome investigated was suboptimal maturation rate. In cases in which no oocyte was retrieved in one ovary and rescue hCG was given, the LH and progesterone levels after the first retrieval and the oocyte number and maturity at the second retrieval were documented.

### Ovarian stimulation and oocyte retrieval

Before starting IVF treatment, the patients were assessed for ovarian reserve parameters, including hormonal profile and/or antral follicle count (AFC), at the early follicular phase. Several patients were also tested for anti-Mullerian hormone (AMH).

We defined patients as having lower ovarian reserve (LOR) if FSH>10 IU/L and/or antral follicular count <5-7 follicles and/or AMH less than the 25th percentile corresponding to their age. Patient performance in prior IVF cycles was also considered. All patients underwent standard ovarian stimulation protocols with GnRH antagonist, with or without PT. The trigger for ovulation consisted solely of a GnRH agonist.

The gonadotropin starting dose was decided according to the patient's age, treatment indication, ovarian reserve parameters, BMI and outcomes in previous IVF cycles (if any). The cycle was monitored by hormonal assays and ultrasonography. Ovulation was triggered when two to three ovarian follicles were at least 17-*18* mm in diameter. Oocyte retrieval was performed approximately 36 hours after GnRH agonist triggering and under the guidance of transvaginal ultrasound.

### Oocyte culture following retrieval

The oocytes were enzymatically denuded 2 hours after retrieval using hyaluronidase (SAGE, Cooper Surgical). In cases of oocyte cryopreservation, all mature oocytes were vitrified according to a standard protocol with vitrification medium (SAGE, Cooper Surgical) on straws (Cryotop, Kitazato) in liquid nitrogen tanks (Maxima MVE REC 3000). In cases in which oocyte fertilization occurred, the oocytes were fertilized by IVF or ICSI and were placed in culture dishes (CultureCoin, ESCO Medical Technologies). The zygotes were cultured in vitro with culture medium (GlobalTotal, LifeGlobal Group) in 5% O_2_ and 5% CO_2_ gas condition incubators (Miri Esco).

### Statistical analysis

All analyses were performed using the SPSS Statistics for Windows software, version 28.0 (IBM Corp., Armonk, NY, USA). Continuous variables are expressed as the mean (±standard deviation) or median (with interquartile range). Categorical variables are expressed as numbers (percentages). Univariate analysis using Student’s t test and the Mann-Whitney U test were used to compare differences in continuous parametric and nonparametric variables, respectively, between the two groups. Additionally, the chi-square test or Fisher’s exact test was used to compare differences in categorical variables between groups.

To adjust for relevant cofounders, multivariate logistic regression analyses were conducted to calculate the odds ratios (ORs) with 95% confidence intervals (CIs). The results with a 2-tailed *p* value <0.05 were considered significant.

## RESULTS

### Patient characteristics

The study included 196 patients treated at our IVF unit between January 2019 and July 2021. All of the patients were treated with a GnRH antagonist protocol triggered with a GnRH agonist.

The study population was divided into two groups: the study group (69 patients), in which PT was administered; and the control group (127 patients), in which no PT was given. Most patients in the study group had CHC or GnRH agonist as PT (47.8% and 34.8%, respectively).

The first and second most common indications for treatment were fertility preservation (34.7% were elective, and 3.6% had medical egg cryopreservation), and preimplantation genetic testing - PGT (26%), respectively. Fertility preservation (elective and medical) was the most common indication in the control group, and PGT was the most common indication in the study group (48.8% and 65.2%, respectively).

Table 1 summarizes the differences in patient characteristics between groups. The patients in the study group did not differ significantly in BMI and LOR status compared to controls (*p*=0.991 and *p*=0.675, respectively). However, patients in the study group were younger compared to the controls (*p*=0.016).

**Table 1:** Baseline Characteristics.

Parameters	All (n = 196)	No-PT (n=127)	PT (n=69)	*p* value
Age (years)^*^	32.56 (4.9)	33.18 (4.75)	31.42 (5.08)	NS^a^
BMI (kg/m^2^)^**^	23.34 (21.25-26.29)	23.14 (21.08-26.64)	23.44 (21.35-26.07)	NS^b^
Low ovarian reserve^***^	23 (11.7%)	14 (11%)	9 (13%)	NS^c^
Indication^***^ Cryopreservation PGT Infertility and others	75 (38.3%) 51 (26%) 70 (35.7%)	67 (52.8%) 6 (4.7%) 54 (42.5%)	8 (11.6%) 45 (65.2%) 16 (23.3%)	*p*<0.001^c^
PT type^***^ CHC GnRH agonist Estrogen supplements GnRH antagonist + Estrogen priming Progesterone supplements			33 (47.8%) 24 (34.8%) 5 (7.2%) 4 (5.8%) 3 (4.3%)	

* Data are expressed as mean (+SD)

** Data are expressed as median (interquartile range)

*** Data are expressed as number (percentage)

a Univariate analysis using Student’s t-test

b Univariate analysis using Mann-Whitney U test

c Chi-square test

### Cycle characteristics and outcomes

Table 2 summarizes the differences in cycle characteristics between groups. Overall, 58.2% of the study population was stimulated with FSH only, and 41.8% was stimulated with FSH+LH preparations (recombinant or human menopausal gonadotropin). A significantly greater number of patients in the study group were treated with combined FSH and LH preparations, whereas a greater number in the control group were treated with FSH-only preparations (*p*=0.031).

**Table 2:** Cycle characteristics.

Parameters	All (n = 196)	No-PT (n=127)	PT (n=69)	*p* value
Protocol type^*^ FSH only FSH + LH	114 (58.2%) 82 (41.8%)	81 (63.8%) 46 (36.2%)	33 (47.8%) 36 (52.2%)	0.031^a^
Maximal E2 (pmol/ml)^**^	9081.5 (5705.25-12944.25)	9338.5 (5894.75-13050.25)	8736.5 (5584-12725.5)	NS^b^
Days of FSH^***^	10.08 (1.62)	9.77 (1.48)	10.64 (1.72)	<0.001^c^
Total FSH dose (IU)^**^	2400 (1650-3300)	2400 (1497.5-3112.5)	2475 (1800-3825)	NS^b^
Basal LH (IU)^**^	3.2 (2.02-4.97)	3.85 (2.8-5.1)	1.8 (0.63-3.2)	<0.001^b^
LH pre-trigger (IU) ^**^	0.8 (0.32-2.1)	1 (0.5-2.4)	0.5 (0.2-1.35)	<0.001^b^
Progesterone pre-trigger (IU)^**^	2.25 (1.4-3.32)	2.2 (1.4-3.2)	2.4 (1.4-3.75)	NS^b^
Number of oocytes retrieved^**^	14 (9-21)	15 (9-21)	12 (8-21.5)	NS^b^
Number of MII oocytes retrieved^**^	11 (6-17)	12 (8-21.5)	9 (5-18)	NS^b^
OMR %^**^	80 (65.88-91.54)	78.95 (66.66-91.66)	82.14 (64.95-91.42)	NS^b^
Re-trigger^*^	5 (2.55%)	0	5 (7.2%)	0.005^d^
Suboptimal response^*^	7 (3.6%)	1 (0.8%)	6 (8.7%)	0.008^d^

MII, metaphase II

* Data are expressed as number (percentage)

** Data are expressed as median (interquartile range)

*** Data are expressed as mean (+SD)

a Chi-square test

b Univariate analysis using Mann-Whitney U test

c Univariate analysis using Student’s t-test

d Fisher's exact test

The median maximal estradiol level during the cycle did not differ significantly between the groups (*p*=0.527). However, the mean number of stimulation days were significantly greater in the study group than in the control group (*p*<0.001).

Median basal (measured at the start of the stimulation) and pre-trigger LH levels were significantly lower in the PT group than in the control group (*p*<0.001 and *p*>0.001, respectively). Pre-trigger progesterone levels did not differ significantly between the groups (*p*=0.265).

As shown in Table 2, medians of 14 (9-21) oocytes and 11 (6-17) M2 oocytes were retrieved per patient. The median oocyte numbers and median mature oocyte numbers did not differ significantly between the groups (0.093 and 0.257, respectively).

### Effect of PT on primary outcome

In 7 cases, 6 in the study group and 1 in the control group, there was a suboptimal response (8.7% *versus* 0.8%, respectively, *p*=0.008). All patients in the study group with suboptimal response were treated with GnRH-agonist as PT.

Five patients in the study group and none in the control group were re-triggered with hCG (*p*=0.005). The median LH levels after the failed retrieval had an inadequate response to the trigger (0.9 IU, 0.65-1.1). The median numbers of oocytes retrieved and matured oocytes post-trigger were 10 (3-17) and 5 (2-14), respectively.

Univariate logistic regression for PT status showed that PT was an independent predictive factor for suboptimal response (*p*=0.023). Several pre-retrieval variables were identified in the univariate analysis as being significantly different between the groups. These variables include age, duration of stimulation, protocol type, basal and pre-trigger LH values.

Multivariate stepwise logistic regression analysis was performed with suboptimal response as the dependent variable and PT type (divided to ‘GnRH-agonist’ and ‘non GnRH-agonist’ compared to no-PT as reference) and the above-mentioned pre-retrieval variables as the independent variables.

The forward selection method was used to include variables with a *p*-value <0.05 in the multivariate model. The logistic regression model for suboptimal response was statistically significant: χ^2^(3) = 27.24, *p*<0.001 (Table 3). The regression showed that PT was an independent predictive factor for suboptimal response (*p*=0.001). The OR of suboptimal response for PT with GnRH agonist was 110.29 (95% CI 8.7-1395.6).

**Table 3:** Multivariate logistic regression analysis (Step 2 results).

Variable	β	Wald	*p*	OR (95% CI)
Pre-treatment type		13.192	0.001	
Non GnRH-agonist PT	-15.093	0.00	0.998	0.00
GnRH agonist PT	4.703	13.192	<0.001	110.29 (8.716, 1395.586)
Duration of stimulation	-0.584	3.804	0.051	0.558 (0310, 1.003)
Constant	0.585	0.048	0.826	1.794

β regression coefficient, OR odd ratio.

## DISCUSSION

In the present study, we demonstrated that PT with GnRH-agonist in antagonist cycles triggered with an agonist negatively affected response to trigger. Patients under the agonist-antagonist protocol had odds ratios of at least 8.7 for suboptimal response compared to no PT. Using CHC as PT was not associated with such a poor response to trigger.

Pre-treatment in IVF cycles has been suggested in the literature to induce the growth of a homogenous follicular cohort and to optimize mature oocyte yield. Hence, PT is being considered in our IVF unit for patients in whom we anticipate poor response and those who have exhibited asynchronous follicular development in prior cycles. Additionally, it is being considered in PGT patients when biopsy scheduling is needed.

In regards to the agonist-antagonist protocol, two versions of the protocol exist. In one, the GnRH-agonist is given to achieve a flare-up effect (Berger *et al*., 2004; Berker *et al*., 2010; Çelik *et al*., 2015; Yang *et al*., 2019a) and, in the other, to cause pituitary suppression before stimulation starts (Fisch *et al*., 2008; Orvieto *et al*., 2020). The latter is the protocol studied and focused on in our study.

The data in the literature regarding the use of GnRH agonist as PT are scarce. This is the first study to address the outcomes of PT with GnRH agonist in GnRH antagonist cycles with agonist triggering. Comparable to the conventional long protocol, in cases of agonist-antagonist protocol, mid-luteal administration of GnRH-agonist achieves suppression of the physiological secretion of pituitary gonadotropins prior to stimulation. Faber *et al*. (1998) were the first authors to present a beneficial effect of mid-luteal suppression with agonists on low response patients. Other studies compared pre-stimulation mid-luteal agonist with cases in which an agonist was continued throughout the follicular phase (Dirnfeld *et al*., 1999; Garcia-Velasco *et al*., 2000; Simons *et al*., 2005). Yet, there were no differences in the reproductive outcomes between the groups in a Cochrane review (Siristatidis *et al*., 2015).

Recently, Orvieto *et al*. (2020) used the stop GnRH-agonist regimen combined with a GnRH-antagonist protocol in poor responders. Patients received hCG as a trigger for ovulation. The results demonstrated significantly larger numbers of follicles developed, retrieved oocytes and top-quality embryos.

Similar to the protocol presented by Orvieto *et al*. (2020), 34.8% of our pretreated patients received mid-luteal suppression with a GnRH agonist prior to gonadotropin stimulation. However, our patients were triggered with GnRH agonists, since oocyte or embryo cryopreservation was planned or due to the risk of developing OHSS. Additionally, our study population was more heterogeneous since we used this protocol not only in poor responders but also to improve follicular synchronization and/or allow for cycle scheduling in normo-responders.

Failure to retrieve any oocytes have been described in up to 3.5% of IVF oocyte retrievals (Castillo *et al*., 2012). The higher prevalence in our study group, 8.7%, when adequate response was expected according to follicular development and measured estradiol values, indicates that the final step of stimulation, the trigger, is impaired. In GnRH-antagonist cycles, triggering with GnRH agonists displaces the antagonist from the pituitary receptors and results in the activation of LH and FSH release. An immediate LH surge after triggering causes final oocyte maturation and ovulation (Fauser *et al*., 2002). In most oocyte retrievals triggered with GnRH agonists, the oocyte yield and maturation rate are comparable to those triggered by hCG (Griesinger *et al*., 2006). However, there is a small subgroup of patients who display suboptimal oocyte yield and maturation rates. These could be explained by altered folliculogenesis, genetic factors involving the LH/hCG or GnRH receptors, pharmacological issues and human error (Castillo *et al*., 2012; Stevenson & Lashen, 2008; Revelli *et al*., 2017). However, the causes of the suboptimal response after GnRH agonist triggering differ from those after hCG triggering. In contrast to hCG triggering, GnRH agonists have a flare-up effect on the pituitary with the release of LH and FSH after activating the GnRH receptors. Thus, a suboptimal response after GnRH agonist administration could represent a state of pituitary dysfunction with insufficient endogenous LH release. In such cases, the endogenous release of LH is insufficient and inadequate (in magnitude and/or duration) to achieve final oocyte maturation. Hence, a suboptimal response to GnRH agonist triggering is associated with different risk factors suggestive of hypothalamic and/or pituitary hypofunction. Some of them include patient characteristics, such as low BMI and the presence of hypothalamic amenorrhea, and others involve cycle characteristics, such as low basal and pre-trigger LH and higher total dosage of gonadotropins required for stimulation (Meyer *et al*., 2015; Chang *et al*., 2016; Lu *et al*., 2016).

Patients with long-term hormonal contraception have also been reported to be at risk for suboptimal response (Popovic-Todorovic *et al*., 2019; Meyer *et al*., 2015; Engmann *et al*., 2016). Low basal and pre-trigger LH after the use of hormonal contraception have been described as markers for an increased risk of suboptimal response to GnRH agonist triggering. Similarly, our study demonstrated lower basal and pre-trigger LH values among patients who received PT. However, none of the patients with CHC as PT demonstrated suboptimal response. It appears that PT with agonist creates longer and deeper suppression on the pituitary compared to CHC or other PT modalities.

Duration of ovarian stimulation was found near statistical significance in the multivariate logistic regression model (*p*=0.051). Longer stimulation period lowers the risk of a suboptimal maturation response. The literature regarding the optimal stimulation duration and impact on IVF outcomes has been conflicting (Chuang *et al*., 2010; Sarkar *et al*., 2019; Yang *et al*., 2019b; Stout *et al*., 2022). In cases of PT with a GnRH agonist, a prolonged stimulation period could supposedly enable a recovery time for the desensitized pituitary. Pituitary recovery after GnRH agonist administration is timeand dose-dependent (Broekmans *et al*., 1996); thus, responsiveness should be expected several days after cessation of agonist administration. Hence, a longer stimulation duration might potentially be a protective factor for pretreated patients who exhibit an adequate maturation response.

Although group size calculation supported the detection of an adverse outcome with 95% confidence and 80% power, our study is limited by the number of patients encompassing the study group (and in each PT type sub-groups). Therefore, we could not determine which factors could accurately differentiate cases of PT with adequate response from those with suboptimal response. Nevertheless, it is possible that a subgroup of patients is more susceptible to deeper and longer pituitary suppression, which lasts beyond the expected recovery point.

Due to the nature of our study population, in which at least 38% of our patients underwent oocyte or embryo cryopreservation, we lack information about implantation and clinical pregnancy rates. As a result, other reproductive outcomes are not within the scope of this study.

## CONCLUSIONS

Pre-treatment with GnRH agonists in the constellation of antagonist protocol cycles triggered with agonists is associated with an increased risk for poor maturation response. It appears that trigger efficacy is compromised since the target organ, the pituitary, remains in deep suppression as shown by the significantly low LH pre-trigger values. Therefore, if PT with GnRH agonist is used with agonist triggering, post-trigger LH measurement should be strongly considered. Accordingly, administration of a low dose or rescue hCG trigger and delaying of oocyte retrieval can be performed.

Nonetheless, large, prospective studies should be conducted to clarify the role of the agonist-antagonist protocol in ART practice and to investigate its efficacy. Additionally, more studies are needed to define specific subgroups at risk for suboptimal response and those who might benefit from PT in general and the agonist-antagonist protocol in particular.
